# Do antenatal preparation and obstetric complications and procedures interact to affect birth experience and postnatal mental health?

**DOI:** 10.1186/s12884-023-05846-5

**Published:** 2023-07-27

**Authors:** Hannah Cross, Charlotte Krahé, Helen Spiby, Pauline Slade

**Affiliations:** 1 Department of Primary Care and Mental Health, Institute of Population Health, University of Liverpool, Liverpool, UK; 2 Lancashire and South Cumbria NHS Foundation Trust, Blackpool, UK; 3 Present Address: School of Psychology, Liverpool John Moores University, Liverpool, UK; 4 School of Health Sciences, University of Nottingham, Nottingham, UK

**Keywords:** Antenatal preparation, Birth experience, Complications, Procedures, Postnatal mental health

## Abstract

**Background:**

Antenatal preparation is commonly offered to women in pregnancy in the United Kingdom, but the content is highly variable, with some programmes orientated towards ‘normal birth’, whilst others may incorporate information about complications and procedures (broader focus). However, the impact of this variability on birth experience has not been explored. We examined the relationship between the content of antenatal preparation received and birth experience, taking into account obstetric complications and procedures. As birth experience can have a profound impact on a mother’s postnatal well-being, we also investigated associations with mothers’ postnatal mood and anxiety.

**Methods:**

*N* = 253 first-time mothers completed a cross-sectional survey measuring demographic and clinical factors, antenatal preparation content (categorised as normality-focused or broader-focused), obstetric complications and procedures experienced, birth experience (measured using three separate indices; the Childbirth Experience Questionnaire, emotional experiences, and presence/absence of birth trauma), postnatal depression and anxiety, and qualitative information on how the COVID-19 pandemic had affected birth experience.

**Results:**

Regarding birth experience, receiving more broader-focused preparation was associated with a more positive birth experience irrespective of complications/procedures experienced, while receiving only normality-focused preparation was beneficial in the context of fewer complications/procedures. Regarding birth trauma, receiving more broader-focused preparation was associated with lower likelihood of reporting birth as traumatic only in the context of more complications/procedures. Degree of normality-focused preparation was unrelated to experience of birth trauma. Lastly, while more complications/procedures were associated with greater anxiety and low mood, only greater normality-focused preparation was linked with better postnatal mental health.

**Conclusions:**

Antenatal preparation including both normality- and broader-focused information is positively related to women’s birth experience. While normality-focused preparation seems most beneficial if fewer complications/procedures are experienced, broader-focused preparation may be most beneficial in the context of a greater number of complications/procedures. As complications/procedures are often unpredictable, offering broader-focused preparation routinely is likely to benefit women’s birth experience. This antenatal preparation should be freely available and easily accessible.

**Supplementary Information:**

The online version contains supplementary material available at 10.1186/s12884-023-05846-5.

## Introduction

Childbirth can be unpredictable, with the possibility of both obstetric complications and procedures [1]. In England and Wales in 2019, an estimated 43% of women giving birth had a caesarean or instrumental birth [2]. A complication is an event occurring during labour and birth that requires assistance from healthcare professionals and that may require obstetric procedures. Procedures can be used for several reasons, often as a result of professionals becoming concerned about the welfare of either mother or baby [3]. Examples of procedures include induction of labour [4], episiotomy, and active management of the third stage of labour to reduce the risk of a postpartum haemorrhage. Both complications and procedures clearly have potential implications for women’s birth experience.

Women who report feeling unprepared for birth through experiencing a discrepancy between their expectations and the actual experience are more likely to report birth as traumatic [5]. A traumatic birth experience can result in women developing postnatal post-traumatic stress disorder, particularly when women experience an unexpected and potentially harrowing obstetric complication or procedure [6]. Molyneux, Fowler and Slade [7] suggested that procedures, such as episiotomy, are associated with a more negative childbirth experience, and can cause physical harm and disruption to women postnatally at a time when they are trying to bond with their newborn [8]. This highlights the importance of understanding the impact of obstetric complications and procedures (OC&P) on women’s birth experience and postnatal mental health.

Pregnant women in the United Kingdom (UK) can access free National Health Service [9] antenatal preparation, often in the form of classes, to give them information and develop coping strategies to help prepare them for the birth of their baby. In a systematic review, Brixval, Axelsen [10] found insufficient evidence to determine whether antenatal education classes were effective in improving obstetric outcomes or birth experiences. However, the focus and content of preparation was varied and non-standardised and was not considered. Whilst guidance outlined by the National Institute for Health and Care Excellence [11] states that pregnant women in the UK should be offered opportunities to attend antenatal preparation classes, the content and number of classes offered is at the discretion of individual NHS Trusts [2]. Provision of antenatal preparation was highlighted as an area requiring improvement in a recent UK maternity survey [2]. Only 71% of women reported being offered NHS antenatal preparation classes and only 30% of women surveyed attended [2, 3]. Redshaw and Henderson [3] noted that 14% of women attended non-NHS privately funded antenatal preparation classes. Access to provision and uptake of antenatal preparation as well as content is therefore highly variable.

Information about the onset of labour, the stages of labour and birth, and breastfeeding are topics routinely covered by most antenatal preparation provisions [12] and are defined in this study as normality-focused preparation. Some antenatal preparations may also cover broader-focused topics, which include information on OC&P possible during childbirth [9, 13]. It is noted that generally women want information from UK maternity services to help them understand procedures that may be used during labour/birth [14]; however, this is the topic found least likely to be included by maternity services [12]. The reasons for this are unclear. Time limitations may be one explanation [15], however, it is also considered that some UK maternity professionals may be concerned that discussing the possibility of birth not proceeding straightforwardly, and the potential for OC&P, may inadvertently raise women’s anxieties or reduce the possibility of ‘normal birth’ [16]. Such concerns are not borne out in other and admittedly different healthcare contexts, where numerous studies investigating the impact of preparatory information have typically found additional information beneficial. For example, preparatory information helped reduce chemotherapy patients’ pre-treatment anxiety and enhanced their satisfaction and confidence in coping with treatment when compared to control patients [17]. Similarly, Mott [18] found a significant difference existed between the pre- and post-procedure anxiety scores overall within a cardiac catheterisation sample. Additional video information was found to increase patient ability to relax during examination in a cardiovascular magnetic resonance imaging sample [19]. Furthermore, anxiety scores were lower in parents of children in a paediatric intensive care unit when provided with information preparing them for their child’s ward transfer [20]. These studies support the idea that preparing individuals for a potentially stressful procedure has a positive impact on their experience and anxiety. This could also be true for women facing OC&P during childbirth, but this possibility has not previously been explored.

Psychological theories such as the transactional theory of emotions, stress, and coping proposed by Lazarus and Folkman [21] would certainly support this premise. They highlight that people appraise information to consider the implications for their wellbeing. An individual’s coping and emotional response to information depends on their appraisal of the perceived harm, threat, or challenge to their wellbeing, particularly if the appraisal is accompanied by anxiety [21]. In line with this model, it can be hypothesised that antenatal preparation which does not include information on potential OC&P during childbirth may lead to a higher appraisal of perceived threat, and therefore more negative birth experience and postnatal mental health.

Understanding whether information on possible OC&P provided during antenatal preparation and actual occurrence of such OC&P during labour relate to birth experience and postnatal mental health could inform the provision of antenatal preparation. The aim of this study was to explore the relationship between the content of antenatal preparation received, the experience of OC&P (as defined by number of complications and procedures, weighted by their severity), and birth experience (measured using three separate indices: the Childbirth Experience Questionnaire (CEQ), emotional experiences, and presence/absence of birth trauma). Furthermore, we explored associations with postnatal mood and anxiety.

### Hypotheses

1. Birth experience would be associated with the experience of OC&P and the type and amount of antenatal preparation. More specifically, receiving broader-focused preparation (information about complications and procedures) would be associated with a more positive birth experience, especially in the context of more OC&P. We expected normality-focused preparation to be positively associated with birth experience in the context of lower levels of OC&P.
2. Postnatal anxiety and low mood would be associated with the experience of OC&P and the type and amount of antenatal preparation. More specifically, receiving more broader-focused preparation would be associated with lower levels of anxiety and depression symptoms, especially in the context of more OC&P. We expected more normality-focused preparation to be positively associated with mood in the context of lower levels of OC&P.

## Method

### Design

This study used a quantitative cross-sectional design. Birth experience, and postnatal anxiety and depression were outcome variables. Amount of antenatal preparation, number of OC&P (weighted by their severity), and their interaction, were predictor variables. Normality-focused and broader-focused preparation were examined separately.

### Participants

First time mothers **(**aged ≥ 18 years of age) who were able to understand English, with babies born 37 + weeks, were invited to participate 4–12 weeks postnatally. Women were eligible if they had attended at least one antenatal class in person or virtually, either NHS or privately funded. Women who had a planned caesarean section, gave birth to more than one baby, whose baby required more than 48 h in special care, or whose baby was stillborn were excluded. All participants were from the UK.

To determine the sample size required, an a priori power analysis was calculated for multiple regression analysis. With an alpha (α) = 0.05 and power = 0.95, the projected sample size needed for a medium effect size *f* = 0.15 (*R*^*2*^ = 0.13) was *N* = 119, assuming two predictors and their interaction (GPower 3.1).

### Procedure

The study was advertised across social media platforms for perinatal women (e.g., Mums Aid, Pandas Foundation, and Birthrights). The survey included questions regarding demographic information, information on antenatal preparation accessed, OC&P experienced, as well as measures of birth experience and measures of postnatal anxiety and depression. The survey was completed anonymously online following the information sheet and completion of the consent form. Recruitment ran from June 2020 to December 2020. Ethical approval was granted by the University of Liverpool Research Ethics Committee on the 07/05/2020.

### Measures

#### Antenatal preparation scale (APS)

A list was developed to measure antenatal preparation information, drawing on a recent survey of antenatal education provision Spiby, Stewart et al. [12]. The list aimed to cover the focus and full range of potential content of antenatal preparation, including both normality- and broader-focused items. Broader-focused items incorporated information about potential complications and procedures. This list was reviewed by attendees of a mother-and-baby group to ensure completeness and ease of comprehension (see supplementary materials 1 for further information).

When completing this scale, women indicated which elements were covered within the preparation they attended. The list included information on aspects usually included in antenatal education, such as signs of labour starting, the three stages of labour, pain relief, breastfeeding, and their wellbeing. In addition, women were asked whether information on possible OC&P was provided (broader-focused items). Women were asked to indicate if they had received “No information” (scored 1), “Some limited information” (scored 2), or “Detailed information” (scored 3) for each item. Where women had attended more than one set of antenatal preparation, they were asked to report on each set, and their highest score of information received within any of the programmes was utilised. Thus, a composite score was created for those who had attended two or more classes, with their highest scores for each item of both normality- and broader-focused information used to provide individual subtotals for the two dimensions. Internal consistency was high for both the normality-focused 18-item scale (α = 0.92; possible scores ranging from 18 to 54) and the broader-focused 22-item scale (α = 0.95; possible scores ranging from 22 to 66).

#### Obstetric complications and procedures scale (OCPS)

To measure experience of OC&P, a list was created to cover the OC&P experienced by mothers, corresponding to the antenatal information measure. As with the APS above, the list was reviewed by attendees of a mother-and-baby group to ensure completeness and ease of comprehension (see supplementary materials 1 for further information).

As a composite measure of OC&P was required, nine midwives in current clinical practice rated the OCPS items on how ‘severe and sudden’ they believed each individual item to be, with 1 being “Not at all severe and sudden”, to 5 being "Extremely severe and sudden". The means of these scores were used to weight each individual item to create a continuous scale utilised in the analysis (see Supplementary Table 1). A total score for each participant was calculated by adding up the individual item weighting for each item selected (for example, breaking waters, rupturing membranes artificially (2) + third- or fourth-degree perineal tear (5) = 7). Higher scores reflect more severe and sudden OC&P during birth (possible scores ranged from 0 to 67). Intraclass correlation coefficients (ICC) were computed to check midwife interrater reliability. Based on a mean-rating (*k* = 9), the two-way random, absolute ICC  0.87. This is indicative of good interrater reliability for this measure [22].

#### Experience of childbirth

##### Childbirth experience questionnaire (CEQ)

The Childbirth Experience Questionnaire (CEQ; [23]) was used to assess general experience of birth, which incorporates questions about experiences of own capacity, professional support, perceived safety, and participation. The CEQ consists of 19 items scored on a four-point scale, with options ranging from 1 “totally disagree” to 4 “totally agree”. Higher ratings reflect more positive experiences (possible total scores range from 19 to 76). The CEQ had excellent internal consistency in this study (Cronbach’s α = 0.91).

##### Overall emotional experience

Section A of Expectations, Experiences and Satisfaction with Labour [24] was used to assess the emotional experience of birth. This section contains 10 questions about emotions during labour, five positive (Exciting, Enjoyable, Satisfying, Pleasant, Exhilarating) and five negative (Anxiety provoking, Frightening, Embarrassing, Exhausting, Difficult) derived from interviews with women in the postnatal period. These items were rated on an on a four-point scale, with options ranging from 1 “not at all” to 4 “extremely”. As expected, the negative emotions factor and the positive emotions factor were significantly negatively correlated (*r* = − .60, *p* < .001) and an overall emotional experience score was generated by reversing the scores for negative emotion items, which were added to the scores for the positive items. Possible scores ranged from 10 to 40, with higher scores indicating an overall more positive experience. The scale demonstrated good internal consistency (α = 0.89).

#### Trauma-related questions

Two questions relating to trauma were used to assess traumatic experience of birth and required a “Yes /No” response. These questions were, “Thinking about your childbirth (and any time in hospital after), was there any time during this when you felt: (i) horror or helplessness about what was happening and (ii) really frightened about your own or your baby’s wellbeing?”. These questions were derived from DSM-IV-R criteria with modifications tailoring them to childbirth from the Birth Trauma Association, and previously used to assess whether childbirth was traumatic in a large post-traumatic stress disorder prevention trial [25]. Women who answered yes to both were considered to have had a traumatic experience of birth (*n* = 97).

#### Measures of postnatal mental health

The Generalised Anxiety Disorder Assessment Scale (GAD-7; [26]) was used to assess anxiety. The GAD-7 contains seven questions and is used in clinical services. Items include, “Feeling nervous, anxious or on edge” and are scored on a four-point scale, with options ranging from "0 (not at all)" to "3 (nearly every day)". Higher total scores reflect higher levels of anxiety (possible scores range from 0 to 21). The GAD-7 demonstrated excellent internal consistency in this study(α = 0.92).

The Patient Health Questionnaire measure of Depression (PHQ-9; [27] was used to assess mood. The PHQ-9 contains nine questions, validated in clinical samples. Items include “Feeling down, depressed, or hopeless” and are scored on a four-point scale, with options ranging from "0 (not at all)" to "3 (nearly every day)". Higher total scores reflect higher levels of depression (possible scores range from 0 to 27). The PHQ-9 demonstrated good internal consistency in this study (α = 0.88).

#### Impact of the COVID-19 pandemic

As recruitment took place during the COVID-19 pandemic, four questions explored the impact of this context on participants’ birth experience. These were, “Did this impact on what antenatal preparation you were able to access?”, “Were you able to have ALL the people you had planned to be with you in labour and birth?”, “If someone was with you for the birth, were they able to be there for all the time you had planned?”, and “Was your birth experience affected in any other way by the COVID pandemic?” (see supplementary materials 1 for additional information).

### Data analysis

Following checks to ensure parametric analysis was appropriate, bivariate associations were examined. To test Hypothesis 1, hierarchical multiple regression analyses were carried out to investigate the impact of antenatal preparation content (separately for normality-focused and broader-focused preparation) alone and in interaction with experience of OC&P on women’s birth experience variables, namely CEQ, and overall emotional experience. OC&P were entered in step 1, antenatal preparation in step 2, and their interaction in step 3. Logistic regression analyses were carried out to examine the relationship between preparation, OC&P, and traumatic experience of childbirth (binary outcome), with steps as above. Significant interactions were plotted at − 1*SD* and + *1SD* of the continuous predictor variables, and significance was tested using the ‘lincom’ command in Stata [28].

To test Hypothesis 2, hierarchical multiple regression analyses considered the impact of antenatal preparation content alone and in interaction with experience of OC&P on postnatal mental health measures of anxiety and depression (steps same as above).

A content analysis was conducted to summarise the brief contextual information provided exploring the impact of the COVID-19 pandemic on participants’ birth experience [29].

### Final sample

In a positive response to recruitment efforts, a total of 253 participants met the inclusion criteria and were included. Table 1 outlines the sample characteristics of the final sample. Birth dates of babies ranged in an 8-month period from April 2020 to November 2020.

**Table 1:** Characteristics

Demographic	*N* (%)
*Age*	
18–24	36 (14.23)
25–31	151 (59.68)
32–38	63 (24.90)
39–45	3 (1.19)
*Marital Status*	
Single	15 (5.93)
Married	127 (50.20)
Cohabitating	109 (43.08)
Prefer not to say	2 (0.79)
*Highest Level of Education*	
GCSE	16 (6.32)
A level/Vocational Qualification	45 (17.79)
Degree/Postgraduate degree	185 (73.12)
Prefer not to say/other	7 (2.77)
*Employment status pre-maternity leave*	
Employed full time or part time	226 (89.33)
Unemployed	7 (2.77)
Self-employed	3 (1.19)
Employed other: Student/homemaker/prefer not to say	17 (6.71)
*Ethnicity* (no information for 3 participants)	
White British and other	245 (96.84)
Mixed/Multiple ethnic groups	3 (1.19)
Black/African/Caribbean/Black British	1 (0.40)
Prefer not to say	1 (0.40)
*Number sets of antenatal preparation*	
One set	194 (76.68)
Two sets	55 (21.74)
Three sets	4 (1.58)
*Type of antenatal preparation*	
NHS provision only	88 (34.78)
Private only provision	123 (48.62)
Both NHS and private provision	32 (12.65)

Of the sample, 119 (47.04%) participants experienced an unassisted vaginal birth. For the remaining women, the most frequent procedure reported was ‘Electronic monitoring of baby throughout labour’ (*n* = 185; 73.12%), followed by ‘Active management of the third stage of labour’ (*n =* 155; 61.26%) and ‘Membrane sweep’ (*n =* 123; 48.62%) (Table 2).

## Results

### Antenatal preparation completed and OC&P experienced

The average normality preparation score was *M *= 38.87 (*SD* = 8.38), with scores ranging from 18 to 54 (see Table 3), with 65.60% of women reporting having had at least some limited information for all elements. In comparison, the average broader-focused preparation score was *M *= 38.95 (*SD* = 10.58), with scores ranging from 22 to 66, and with only 28.10% of women reporting having had at least some limited information for all elements. This suggested fewer women had received coverage of broader-focused areas of information. It was noted that the time at which the survey was completed by women was unrelated to birth experience (Spearman’s rho = 0.0003, *p* > .005) or emotional experience (Spearman’s rho = − 0.038, *p* > .005). Therefore, we did not control for this factor in our analyses.

**Table 2:** Obstetric complications and procedures

	*N* (%)
Breaking waters artificially	106 (41.90)
Waters breaking a prolonged period before labour or contractions	66 (26.09)
Membrane sweep(s)	123 (48.62)
Gel or pessary	94 (37.15)
Oxytocin drip	85 (33.60)
Augmentation	44 (17.39)
Forceps	42 (16.60)
Ventouse	34 (13.44)
Episiotomy	80 (31.62)
1st or 2nd degree perineal tear	102 (40.32)
3rd or 4th degree perineal tear	14 (5.53)
Breech	8 (3.16)
Nuchal cord	38 (15.02)
Baby distressed during labour	114 (45.06)
Electronic monitoring of baby throughout labour	185 (73.12)
Emergency Caesarean section	71 (28.06)
Active management of the third stage of labour	155 (61.26)
Retained placenta	8 (3.16)
Need for special care baby unit	19 (7.51)
Excessive blood loss after birth	66 (26.09)
Extended stay in hospital for mother 3+ days	45 (17.79)

**Table 3:** Correlations between variables (*N* = 253)

Variable	1	2	3	4	5	6	7	8	*M*	*SD*	
1 Norm Prep	--								38.87	8.38	
2 Broad Prep	0.80 **	--							38.95	10.58	
3 Total Prep	0.94 **	0.96 **	--						77.81	17.99	
4 OCPS	− 0.07	− 0.05	− 0.06	--					16.80	8.50	
5 CEQ	0.20 **	0.19 **	0.20 **	− 0.39 **	--				11.47	2.46	
6 Overall emotional experience	0.20**	0.22**	0.22**	− 0.39 **	0.76**	--			22.67	7.01	
7 Depression	− 0.16 **	− 0.08	− 0.13 *	0.17 **	− 0.36 **	− 0.42 **	--		6.85	5.70	
8 Anxiety	− 0.13*	− 0.08	− 0.11	0.16 **	− 0.35 **	− 0.39 **	0.77 **	--	6.51	5.57	

*Note*: Norm Prep = Normality-focused preparation, Broad Prep = Broader-focused preparation, Total Prep = Total antenatal preparation, OCPS = complications/procedures experienced, CEQ = birth experience, * *p* < .05, ** *p* < .01, (2-tailed).

The average score on the OCPS was *M* = 16.80 out of a possible 64 (*SD* = 8.50, with scores ranging from 0 to 42), indicating that a range of OC&P were experienced by this sample.

Means and bivariate correlations are presented in Table 3. Hypothesis 1 predicted that birth experience would be associated with the experience of OC&P and the type and amount of antenatal preparation. Considering the first element of this hypothesis, the CEQ and the emotional measure of birth experience were both found to be significantly negatively correlated with the experience of OC&P (OCPS), *r* = − .39, *p* < .01 and *r* = − .39, *p* < .01, respectively, meaning that a more positive birth experience was associated with fewer OC&P, supporting Hypothesis 1. Furthermore, more OC&P were associated with greater anxiety symptoms (*r* = .16, *p* < .01) and depression symptoms ( = .17, *p* < .01; see Table 3). Both the CEQ and overall emotional experience were positively correlated with normality-focused preparation, *r* = .20, *p* < .01, and *r* = .20, *p* < .01, respectively, and broader-focused preparation, *r* = .19, *p* < .01, and *r* = .22, *p* < .01, respectively (see Table 3).

### Antenatal preparation and OC&P interact to predict birth experience (CEQ and overall emotional experience)

#### Normality-focused preparation

##### CEQ

**Fig. 1:**
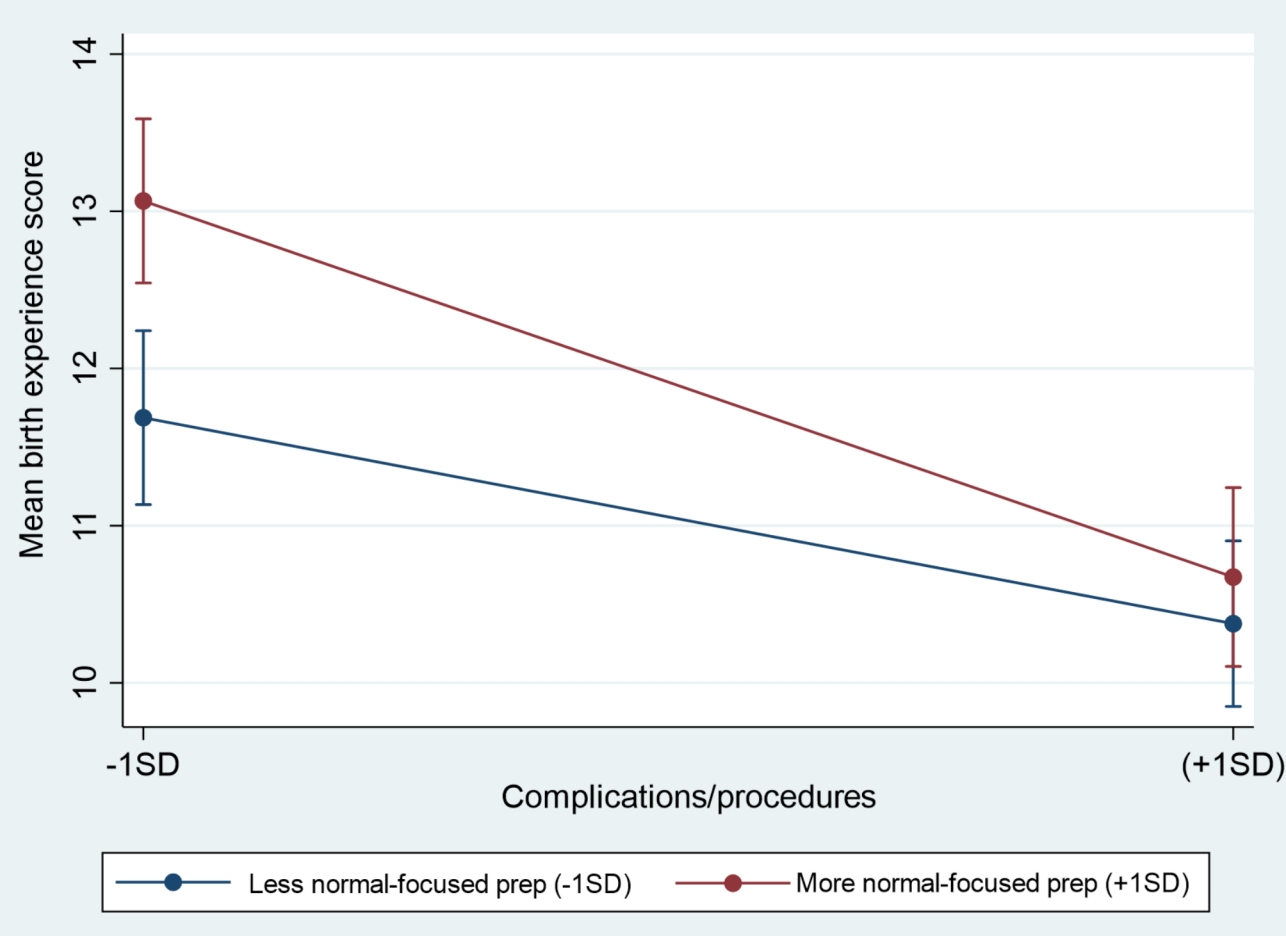
Interaction between complications/procedures and normality-focused preparation on birth experience, plotted at − 1*SD* and + 1*SD* of the scores. Error bars denote 95% confidence intervals

In the regression analysis, fewer OC&P (β = − 0.37, *p* < .001) and greater normality-focused preparation (β = 0.18, *p* = .003) were associated with a more positive birth experience (see Table 4). Including the interaction between the two predictors in step 3, the model accounted for 19% of the variance in birth experience and revealed a significant interaction effect (β = − 0.55, *p* = .039; see Fig. 1). The interaction indicated that more normality-focused preparation was beneficial in the context of fewer OC&P (*p* < .001) but not more OC&P (*p* = .448), supporting Hypothesis 1.

**Table 4:** Summary of the hierarchical regression models for normality-focused preparation

Birth Experience				Overall Emotional Experience			
Variable	B	SE B	β	Variable	B	SE B	β
Step 1				Step 1			
Constant	13.35	0.32		Constant	28.13	0.90	
Com/Pro	− 0.11	0.02	− 0.39***	Com/Pro	− 0.33	0.05	− 0.39***
Step 2				Step 2			
Constant	11.30	0.75		Constant	22.18	2.11	
Com/Pro	− 0.11	0.02	− 0.37***	Com/Pro	− 0.32	0.05	− 0.38***
Normality Prep	0.05	0.02	0.18**	Normality Prep	0.15	0.05	0.18**
Step 3				Step 3			
Constant	8.85	1.39		Constant	22.48	3.97	
Com/Pro	0.04	0.07	0.13	Com/Pro	− 0.33	0.21	− 0.40
Normality Prep	0.11	0.03	0.39**	Normality Prep	0.14	0.10	0.17
Com/Pro * Normality Interaction	− 0.00	0.00	− 0.55*	Com/Pro * Normality Interaction	0.00	0.01	0.02
*R*² = 0.15 for step 1 (*p* < .001), ∆*R*² = 0.03 for step 2, ∆*R*² = 0.01 for step 3				*R*² = 0.16 for step 1 (*p* < .001), ∆*R*² = 0.03 for step 2, ∆*R*² = 0.00 for step 3			

*Note.* Com/Pro = Complications /Procedures. *** *p* < .001; ** *p* < .01; * *p* < .05, Change in *R*² (denoted as ∆*R*²)

##### Overall emotional experience

Overall emotional experience during birth was significantly more positive for women experiencing fewer complications procedures (β = − 0.38, *p* = .000) and greater normality-focused preparation (β = 0.18, *p* = .002; see Table 4). However, the interaction between OC&P and normality-focused preparation was not significant for overall emotional experience, failing to support Hypothesis 1.

#### Broader-focused preparation

##### CEQ

In the regression analysis, fewer OC&P (β = − 0.38, *p* = .000) and more broader-focused preparation (β = 0.17, *p* = .004) were associated with a more positive birth experience (see Table 5). However, the interaction between the two in step 3 was not significant, suggesting that more broader-focused preparation was associated with more positive birth experience irrespective of the number of complications experienced, and that higher levels of OC&P were associated with a less positive birth experience irrespective of the amount of broader-focused preparation received. These findings thus did not support Hypothesis 1.

**Table 5:** Summary of the hierarchical regression models for broader-focused preparation

Birth experience				Overall emotional experience			
Variable	B	SE B	β	Variable	B	SE B	β
Step 1				Step 1			
Constant	13.35	0.32		Constant	28.13	0.90	
Com/Pro	− 0.11	0.02	− 0.39***	Com/Pro	− 0.33	0.05	− 0.39***
Step 2				Step 2			
Constant	11.79	0.62		Constant	22.92	1.74	
Com/Pro	− 0.11	0.02	− 0.38***	Com/Pro	− 0.32	0.05	− 0.39***
Broader Prep	0.04	0.01	0.17**	Broader Prep	0.13	0.04	0.20**
Step 3				Step 3			
Constant	11.06	1.12		Constant	25.87	3.15	
Com/Pro	− 0.06	0.06	− 0.22	Com/Pro	− 0.50	0.17	− 0.61**
Broader Prep	0.06	0.03	0.25*	Broader Prep	0.06	0.08	0.09
Com/Pro * Broader Interaction	− 0.00	0.00	− 0.18	Com/Pro * Broader Interaction	0.01	0.00	0.26
*R*² = 0.15 for step 1 (*p* < .001), ∆*R*² = 0.03 for step 2, ∆*R*² = 0.00 for step 3				*R*² = 0.16 for step 1 (*p* < .001), ∆*R*² = 0.04 for step 2, ∆*R*² = 0.00 for step 3			

*Note.* Com/Pro = Complications /Procedures. *** *p* < .001; ** *p* < .01; * *p* < .05, Change in *R*² (denoted as ∆*R*²)

##### Overall emotional experience

Emotional experience of birth was significantly more positive in the context of fewer OC&P (β = − 0.39, *p* = .000; see Table 5) and more broader-focused preparation (β = 0.20, *p* = .001); again, the interaction was not significant. Therefore, more antenatal preparation, whatever its focus, and lower levels of OC&P were associated with a more positive emotional experience of birth; this part of Hypothesis 1 was not supported (see supplementary materials for further information on Total preparation supplementary Tables 2 and 3).

### Traumatic birth

Of the sample, 38.3% answered yes to both trauma questions, fulfilling the trauma criterion. These women reported less normality-focused, less broader-focused and thus less total antenatal preparation overall (see Supplementary Table 4 for differences between traumatic and non-traumatic birth groups). Women experiencing traumatic birth also experienced significantly more OC&P.

#### Normality-focused preparation

The logistic regression analyses showed that OC&P were significantly associated with trauma (OR = 1.12, *p* = .000; Table 6). However, neither normality-focused preparation nor the interaction between this and OC&P were significant (Table 6). Thus, this part of Hypothesis 1 was not supported.

**Table 6:** Summary of the logistic regression for the trauma-related questions

Trauma experienced/Normality-focused preparation					Trauma experienced/Broader-focused preparation				
Variable	B (SE)	95% CI for odds ratio			Variable	B (SE)	95% CI for odds ratio		
		Lower	Odds ratio	Upper			Lower	Odds ratio	Upper
Step 1					Step 1				
Constant	− 2.38 (0.36)				Constant	− 2.38 (0.36)			
OCPS	0.11*** (0.02)	1.08	1.12	1.16	OCPS	0.11*** (0.02)	1.08	1.12	1.16
Step 2					Step 2				
Constant	− 1.25 (0.74)				Constant	− 1.07 (6.30)			
OCPS	0.11*** (0.02)	1.07	1.12	1.16	OCPS	0.11 ***(0.02)	1.08	1.12	1.16
Normality Prep	− 0.03 (0.02)	0.94	0.97	1.00	Broader Prep	− 0.04 * (0.01)	0.94	0.97	0.99
Step 3					Step 3				
Constant	− 1.89 (1.70)				Constant	− 3.59 (1.41)			
OCPS	0.15 (0.09)	0.97	1.16	1.39	OCPS	0.26 ** (0.08)	1.11	1.30	1.51
Normality Prep	− 0.01 (0.04)	0.91	0.99	1.07	Broader Prep	0.03 (0.03)	0.96	1.03	1.10
OCPS * Normality Interaction	− 0.00 (0.00)	1.00	1.00	1.00	OCPS * Broader Interaction	− 0.00 * (0.00)	0.99	1.00	1.00
*R*² = 0.03 (Hosmer & Lemeshow), 0.15 (Cox & Snell), 0.21 (Nagelkerke). Model X^2^(1) = 8.77 *R*² = 0.04 (Hosmer & Lemeshow), 0.16 (Cox & Snell), 0.22 (Nagelkerke). Model X^2^(1) = 11.16, for step 2 *R*² = 0.04 (Hosmer & Lemeshow), 0.16 (Cox & Snell), 0.22 (Nagelkerke). Model X^2^(1) = 10.76, for step 3					*R*² = 0.03 (Hosmer & Lemeshow), 0.15 (Cox & Snell), 0.21 (Nagelkerke). Model X^2^(1) = 8.77 *R*² = 0.02 (Hosmer & Lemeshow), 0.17 (Cox & Snell), 0.24 (Nagelkerke). Model X^2^(1) = 6.35, for step 2 *R*² = 0.02 (Hosmer & Lemeshow), 0.19 (Cox & Snell), 0.25 (Nagelkerke). Model X^2^(1) = 6.91 for step 3				

*** *p *< .001; ** *p* < .01; * *p* < .05

#### Broader-focused preparation

**Fig. 2:**
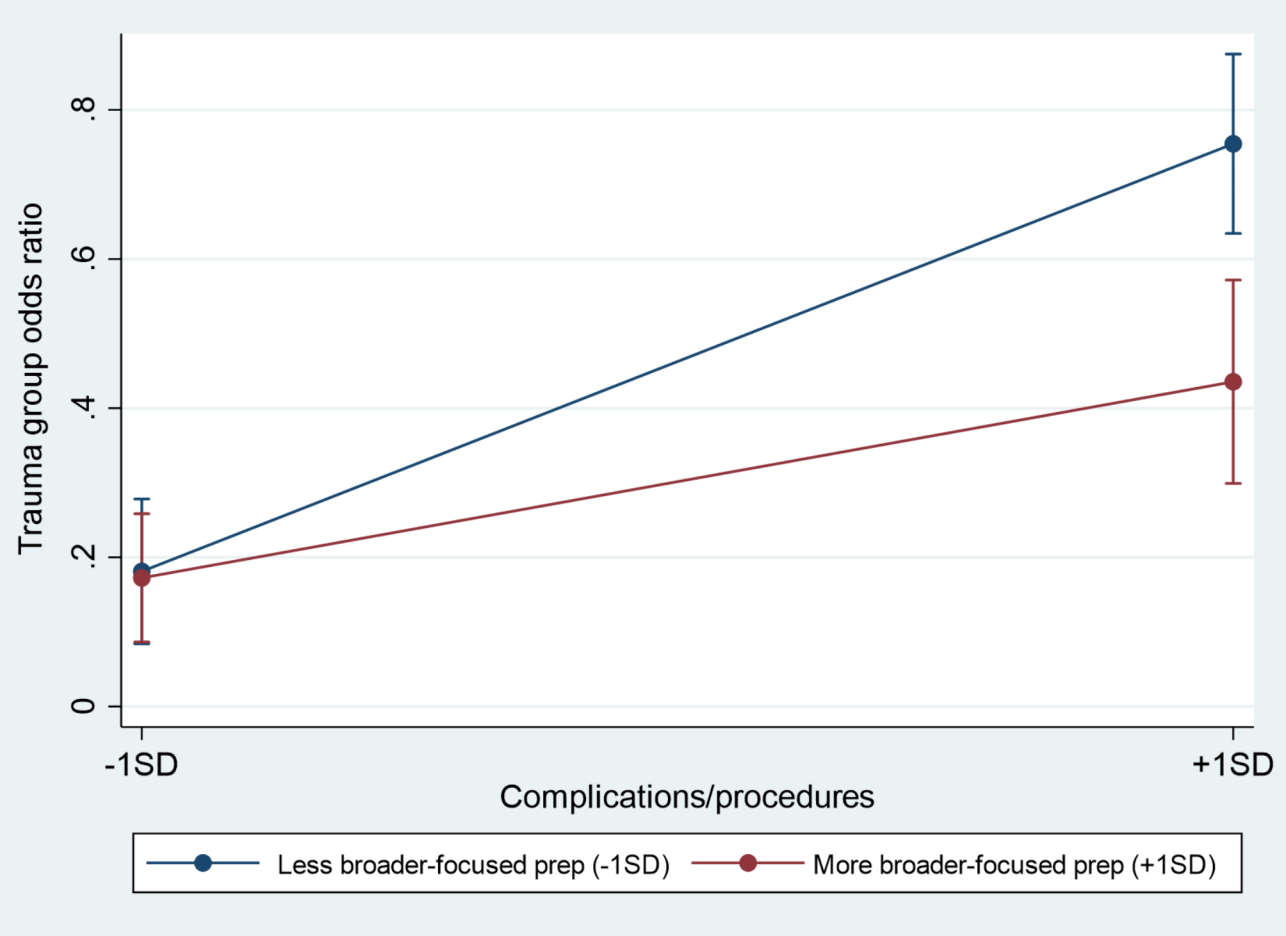
Interaction between complications/procedures and broader-focused preparation on trauma group odds ratio, plotted at − 1*SD* and + 1*SD* of the scores. Error bars denote 95% confidence intervals

OC&P were significantly associated with traumatic birth experience (OR = 1.12, *p* = .000). The interaction between OC&P and broader-focused preparation also revealed a significant interaction (the model accounted for an additional 2% of variance in trauma experience). Following up this interaction showed that greater amount of broader-focused preparation was associated with lower odds of experiencing birth trauma only in the context of more (*p* = .001) and not fewer (*p* = .892) OC&P, supporting Hypothesis 1 (Fig. 2). See supplementary Table 5 for further information on total preparation.

### Postnatal mental health

Receiving less normality-focused preparation was associated with greater depression (β = − 0.15, *p* = .014), but not anxiety symptoms, and amount of broader-preparation received was not associated with either depression or anxiety symptoms (see Table 7). Furthermore, none of the interactions between preparation and OC&P were significant; therefore, Hypothesis 2 was not supported.

**Table 7:** Summary of the hierarchical regression models for depression and anxiety outcomes

Depression/Normality-focused preparation				Depression/Broader-focused preparation			
Variable	B	SE B	β	Variable	B	SE B	β
Step 1				Step 1			
Constant	4.92	0.79		Constant	4.92	0.79	
OCPS	0.12	0.04	0.17**	OCPS	0.12	0.04	0.17**
Step 2				Step 2			
Constant	9.09	1.85		Constant	6.54	1.55	
OCPS	0.11	0.04	0.16*	OCPS	0.11	0.04	0.17**
Normality Prep	− 0.10	0.04	− 0.15*	Broader Preparation	− 0.04	0.03	− 0.08
Step 3				Step 3			
Constant	11.73	3.48		Constant	6.90	2.81	
OCPS	− 0.05	0.18	− 0.08	OCPS	0.09	0.15	0.13
Normality Prep	− 0.17	0.09	− 0.25*	Broader Prep	− 0.05	0.07	− 0.09
OCPS * Normality Interaction	0.00	0.01	0.26	OCPS * Broader Interaction	0.00	0.00	0.04
*R*² = 0.03 for step 1 (*p* < .01), ∆*R*² = 0.02 for step 2, ∆*R*² = 0.01 for step 3				R² = 0.03 for step 1 (*p* < .01), ∆*R*² = 0.01 for step 2, ∆*R*² = 0.00 for step 3			
**Anxiety/Normality-focused preparation**				**Anxiety/Broader-focused preparation**			
**Variable**	**B**	**SE B**	**β**	**Variable**	**B**	**SE B**	**β**
Step 1				Step 1			
Constant	4.70	0.77		Constant	4.70	0.77	
OCPS	0.11	0.04	0.16**	OCPS	0.11	0.04	0.16**
Step 2				Step 2			
Constant	7.92	1.82		Constant	6.21	1.52	
OCPS	0.10	0.04	0.16*	OCPS	0.11	0.04	0.16*
Normality Prep	− 0.08	0.04	− 0.12	Broader Prep	− 0.04	0.03	− 0.07
Step 3				Step 3			
Constant	12.36	3.41		Constant	6.07	2.75	
OCPS	− 0.17	0.18	− 0.25	OCPS	0.11	0.15	0.17
Normality Prep	− 0.19	0.09	− 0.29*	Broader Prep	− 0.03	0.07	− 0.07
OCPS * Normality Interaction	0.01	0.00	0.44	OCPS * Broader Interaction	0.00	0.00	− 0.02
*R*² = 0.03 for step 1 (*p* < .01), ∆*R*² = 0.01 for step 2, ∆*R*² = 0.01 for step 3				*R*² = 0.03 for step 1 (*p* < .01), ∆*R*² = 0.00 for step 2, ∆*R*² = 0.00 for step 3			

*Note.* Com/Pro = Complications/Procedures. ** *p* < .01; * *p* < .05, Change in *R*² denoted as ∆*R*²

### Impact of the COVID-19 pandemic

The summary of the themes reported by women on the impact of the pandemic on birth experience is presented in Supplementary materials (see also Supplementary Table 6 and Supplementary Figure 1.

## Discussion

This study explored the impact of content of antenatal preparation and the experience of OC&P on birth experience and postnatal mental health.

The findings indicate that women receiving a greater quantity of information during antenatal preparation, whatever its focus in content, appeared to have a more positive general birth experience and emotional experience. More normality-focused preparation was positively related to birth experience in the context of lower OC&P. Additionally, receiving more broader-focused information (including details on possible OC&P, whether these were later experienced or not) was linked to more positive birth experience and lower likelihood of experiencing the birth as traumatic in the context of experiencing more (as compared to fewer) complications/procedures. Receiving information about OC&P antenatally, even when these did not subsequently occur, was therefore associated with more positive outcomes.

The findings are in line with the National Maternity Review [15] that found women want to be able to access information and be better informed about any risks when pregnant to help empower them in their decision making during childbirth. However, this is the first time the benefits of antenatal preparation have been clearly demonstrated, taking into account women’s experiences of OC&P. Consistent with the transactional theory on emotions, stress, and coping [21], the current study highlights that one process by which antenatal preparation might impact birth experience is by women being provided with broader-focused information. This preparation supports them to make cognitive appraisals during labour regarding any threats or challenges presented by OC&P in a way that is positively associated with their birth experience (coping) and emotional response.

The findings linked to mental health outcomes showed that while OC&P were related to increased anxiety and low mood, antenatal preparation had limited associations with postnatal mental health. The study recruitment coincided with the COVID-19 pandemic and this context will have impacted participants’ experiences of both antenatal preparation and birth experience [30].

Only a third of women in the current sample reported gaining their antenatal preparation from NHS provision only, which is in line with the figures reported in the maternity survey over the past five years [2, 3]. Interestingly, nearly half of this current sample reported attending non-NHS privately funded antenatal preparation, a much higher rate than the 14% previously noted [3]. However, the impact of the pandemic on NHS antenatal provision and consequently these figures is acknowledged, with many women in the current study reporting having no option but to access private provision due to cancelled NHS classes.

In the current sample, women reported having received more information on normality-focused than broader-focused preparation. Over half of the sample reported having had at least some information on all normality-focused items. However, less than a third of the sample reported, on average, having received at least limited information on all broader-focused items. This is in line with previous findings that reflect topics most commonly included in antenatal education [12]. The findings suggest that this gap in typical provision may seriously disadvantage women in terms of not preparing them for the potential experience of complications and procedures with potential for more negative birth experiences [14].

Overall, the proportion of women experiencing OC&P in the current sample was generally representative of the rate reported in recent large-scale maternity surveys [2, 3]. However, the number of women having an unassisted vaginal birth in the current sample (47%) was lower than the figure reported in a recent UK maternity survey (57%; [2]). This may be explained by the current sample being first-time mothers, who are more likely to have an operative or instrumental birth than women who have given birth before [3]. OC&P are also likely to have increased during the pandemic due to several factors including lack of companionship during birth [31].

The greater the quantity of normality-focused antenatal preparation a woman had accessed, the greater the quantity of broader-focused information she had also received. Quantity of either type of information was not associated with actual OC&P experienced; however, both were positively associated with more positive birth experience (CEQ). Together, this information suggests that either women accessed a great deal of information when pregnant about their upcoming childbirth, receiving both normality- and broader-focused preparation, or very limited information across both types of provision. These findings may link to the evidence suggesting individuals can either be information seekers, searching out information and focusing on health threats about their upcoming labour, or they are hypothesised to be information avoiders, avoiding information if they fear that paying attention to it could cause discomfort or distress [32, 33]. In line with the transactional theory on emotions, stress, and coping [21] attending antenatal preparation may be a way of coping, gaining information in an attempt to reduce any stressors linked to their pending childbirth [34]. The current findings dispute any negative associations in terms of birth experience of provision of information, even when not subsequently directly relevant.

It is important to acknowledge the context of this research, which was conducted during the COVID-19 pandemic, and the significant impact this will have had on the women taking part in this study and accordingly our results. At the start of the COVID-19 pandemic, substantial changes were made to the provision of maternity services, including reducing antenatal appointments and preparation offered, and restrictions around birth settings and birth partners [35]. This was reflected in the themes identified from additional information provided by participants about the profound impact the pandemic had on their birth experiences, with the majority (90%) noting their antenatal preparation had been negatively impacted in some way. While over three quarters of women reported having online antenatal preparation instead of face-to-face provision, they were still able to access some antenatal preparation which suggests these findings may be generalisable to a post-pandemic maternity system. Particularly at the beginning of the pandemic, restrictive practices were imposed, possibly to the detriment of women, to promote wider public health [36]. These restrictive practices involved denying women many choices including those around birth partners, with such restrictions suggested to have resulted in increased OC&P [31].

### Strengths and limitations

Given the limited research previously exploring this area, continuous scales not previously validated were created for antenatal preparation and for OC&P experienced by women, which may be a limitation of the study. It is noted that the APS relied upon women’s self-report of their experienced antenatal preparation which is subjective and could not be verified with course providers, and so there could be a discrepancy between the actual content and women’s recall, which is a limitation of this study. While the new scales merit further exvalidation, both yielded high Cronbach’s alpha score suggesting scale reliability. In addition, both scales were developed with Patient and Public Involvement and expert midwifery involvement. Furthermore, the intraclass correlation coefficient highlighted good interrater reliability of the OCPS scale.

In this sample, women’s reporting of current mental health difficulties was highly correlated with birth experience (CEQ). As measures of mental health were not obtained antenatally due to the study being cross sectional, pre-existing mental health difficulties could not be controlled for in the analysis, and the direction of causality of results cannot be ascertained. It is possible that mental states during pregnancy may have influenced access to antenatal preparation. It is also possible that some aspects of broader-focused antenatal preparation, whilst having a positive association with birth experience, did lead to raised anxiety in pregnancy. The cross-sectional design meant this was not assessed. Therefore, it is suggested that future research would benefit from focusing on women’s mental health outcomes prospectively from pregnancy to the postnatal period.

The required sample size of the current study was exceeded, which means that the analyses were appropriately powered. The limited demographic diversity within the current sample is noted, with the majority identifying as white, having a degree or postgraduate degree, stating they were employed on a full-time basis, being married or cohabitating and being aged 25–31, which may suggest a sample bias and thus is a limitation. However, similar majorities within sample demographics have been noted in respondent characteristics of national maternity surveys [3, 37]. The current study relied solely on online recruitment due to the nature of the pandemic, which may also be a limitation. National maternity surveys include both primiparous and multiparous women and utilise random sampling, recruited by the Office for National Statistics using the birth registration records [37]. Although the sampling of the current study relied on opportunistic sampling, many characteristics are comparable with previous maternity reviews, suggesting results may be generalisable.

### Clinical implications

Accessing antenatal preparation was linked to a more positiveand less traumatic birth experience, which highlights that all pregnant women should actively be offered this preparation in the UK, and it should be easily accessible. Receiving more information on broader-focused topics such as OC&P was found to be helpful, irrespective of women’s subsequent birth experience. This suggests this information should be routinely incorporated into all current antenatal preparation.

### Future research

Future psychological research could test for psychological mediating factors, such as women’s appraisal of the perceived harm, threat or challenge during childbirth [21]. This should be completed within a path analysis of the antenatal to postnatal journey, measured at different time points, with sampling aiming to reflect the diversity of the population. This would allow for further longitudinal exploration of the relationship between antenatal preparation, OC&P, and postnatal mental health.

### Conclusion

Antenatal preparation information is positively associated with birth experience, irrespective of women’s experience of OC&P, and should be freely available and easily accessible, covering not just normality-focused information but potential obstetric complications and procedures as well. This is likely to be of benefit to women’s birth experience and may have positive implications for depressive symptoms postnatally.

## Electronic supplementary material

Below is the link to the electronic supplementary material.

Supplementary Material 1

## Data Availability

The datasets used and analysed during the current study available from the corresponding author on reasonable request.

## List of abbreviations

OC&P: Obstetric complications and procedures
CEQ: Childbirth experience questionnaire
CQC: Care Quality Commission
GAD-7: Generalized Anxiety Disorder Scale 7
NICE: The National Institute for Health and Care Excellence
NHS: National Health Service
OCPS: Obstetric Complications and Procedures Scale
PHQ-9: Patient Health Questionnaire 9
UK: United Kingdom
