# Supplementary material for: Do antenatal preparation and obstetric complications and procedures interact to affect birth experience and postnatal mental health?
Source: BMC Pregnancy Childbirth. 2023 Jul 27;23:543. doi: 10.1186/s12884-023-05846-5 (PMC10375777; doi:10.1186/s12884-023-05846-5)
Supplement: Supplementary file 1 — Supplementary Material 1 [file 12884_2023_5846_MOESM1_ESM.docx]

**Supplementary materials**

**Antenatal Preparation Scale (APS)**

The following questionnaire asks a series of questions about any antenatal preparation classes you attended, either face-to-face or online and the information provided. This will include NHS classes, any paid for classes or any other source of antenatal preparation. If you attended multiple classes, either face-to-face or online, please ensure that when you are answering about each type of class, for example NHS or private, that you only tick if the information was provided by that specific class. For example, NHS classes may have covered different information regarding labour and birth than private classes so it is important this is reflected in your answers

| How many different sets of antenatal preparation classes did you go to? (Options 1/2/3/4) | | | |
| --- | --- | --- | --- |
| Please list what these antenatal preparation classes were (e.g. NHS, NCT, etc):  (1)……………………………  (2)…………………………….  (3)…………………………….  (4)……………………………. | | | |
| Thinking about the first set of classes you attended  Who provided this? ……………………………….. | | | |
| Thinking about the first set of classes you attended  How much time did you spend in these classes, please state in your own words e.g. 3 x2 hour sessions  ……………………………………………………………………………………………………………………………………………………………………………………………………………………………………………………………………………………………………………………………………………………………………………………………………………………………………………………………………………………………………………………………………………………… | | | |
| Thinking about the first set of classes you attended were they carried out face-to-face or via an online resource?  Face-to-face/ Online (*Please select*) | | | |
| Did you have to pay for these classes Yes / No *(Please Tick)*  If yes, how much did you pay?  …………………………………………………………………………………………………………………………………………………………………………………………………………………………………………………………………………………………………………………………………………………………………… | | | |
| If you attended classes before giving birth to your baby, how much information were you provided with during your antenatal preparation classes on the following:  ***Please tick either choice 1, 2 or 3 for each question*** | | | |
|  | 1. No information | 2. Some limited information | 3. Detailed information |
| **Before birth** | | | |
| **Q1.** [**Health in pregnancy**](about:blank) **including;**[**healthy diet**](about:blank)**, pelvic floor exercises and signposted to exercise groups/ relaxation classes** |  |  |  |
| **Q2.** [**Emotions and feelings during pregnancy**](about:blank) |  |  |  |
| **Q3. The signs of labour starting** |  |  |  |
| **Q4. What to do if waters break before going into labour** |  |  |  |
| **Stages of labour** | | | |
| Q5. First stage of labour, cervix gradually open up (dilating), When to contact your midwives, Monitoring your baby in labour |  |  |  |
| Q6. Second stage of labour; Finding a position to give birth in, pushing your baby out, what happens when your baby is born |  |  |  |
| Q7. Third stage of labour; after birth management in the delivery of the placenta, active management (when you have treatment to speed things up) and/or physiological management (when you have no treatment and this stage happens naturally) |  |  |  |
| Q8. Positions of birth |  |  |  |
| **Coping with pain in labour** | | | |
| Q9. Coping methods; Relaxation techniques, breathing techniques, use of aromatherapy, use of massage and use of water |  |  |  |
| Q10. The use of gas and air – keep normality |  |  |  |
| Q11. Pain relief by injection (pethidine and morphine) – broader focused |  |  |  |
| **Breaking waters** | | | |
| Q12. Breaking waters, rupturing membranes artificially |  |  |  |
| Q13. Waters breaking (naturally or artificially) a prolonged period before going into labour or having contractions |  |  |  |
| Q14. Induction of labour, membrane sweep(s) |  |  |  |
| **Q15. Induction of labour, use of vaginal gel or pessary** |  |  |  |
| **Q16. Induction of labour, use of oxytocin drip** |  |  |  |
| **Q17. Speeding up labour/ Augmentation** |  |  |  |
| **Types of birth** | | | |
| **Q18. Unassisted vaginal** |  |  |  |
| **Q19. Assisted birth using forceps** |  |  |  |
| **Q20. Assisted birth using ventouse (method of assisting delivery of a baby using a**[**vacuum**](about:blank) **device)** |  |  |  |
| **Q21. Planned caesarean section** |  |  |  |
| **Q22. Emergency caesarean section** |  |  |  |
| **Q23. Water birth** |  |  |  |
| **During Birth** | | | |
| **Q24. Use of an epidural and the process** |  |  |  |
| Q25. Episiotomy (surgical cut in the perineum to enlarge the opening for the baby to come out) |  |  |  |
| Q26. First- or second-degree perineal tears of the skin and other soft tissue around the vagina. |  |  |  |
| Q27. Third- or fourth-degree perineal tear (deeper tears of the skin around the vagina extending to the muscle that controls the anus (the anal sphincter)). |  |  |  |
| Q28. Baby presenting by breech (bottom first) |  |  |  |
| Q29. Nuchal cord (cord round baby’s neck) |  |  |  |
| Q30. Potential for baby to be distressed during labour |  |  |  |
| Q31. Monitoring of baby throughout labour |  |  |  |
| Q32. [Emotions and feelings during](about:blank) birth |  |  |  |
| **After the birth** | | | |
| Q33. Retained placenta, the placenta not coming away without help to remove it |  |  |  |
| Q34. The possibility of the need for special care baby unit |  |  |  |
| Q35. Excessive blood loss after birth |  |  |  |
| Q36. The possibility of the need for an extended stay in hospital for you following labour |  |  |  |
| Q37. Your health after the birth including pelvic floor exercises |  |  |  |
| Q38. Breastfeeding |  |  |  |
| Q39. Bottle feeding |  |  |  |
| Q40. [Emotions and feelings after](about:blank) birth |  |  |  |

The same set of questions were repeated for each antenatal class identified.

| Were you offered antenatal preparation from any other source? Yes / No *(Please tick)* |
| --- |

| **Final question** - Which of your antenatal classes if any did you find most beneficial? Please state and give further information  …………………………………………………………………………………………………………………………………………………………………………………………………………………………………………………………………………………………………………………………………………………………………… |
| --- |

**Scoring**

*Normality focused 18 items* – To calculate this scale add up all normality focused items:

1, 2, 3, 4, 5, 6, 7, 8, 9, 10, 18, 23, 31, 32, 37, 38, 39, 40

*Broader focused 22 items* – To calculate this scale add up all broader focused items;

11, 12, 13, 14, 15, 16, 17, 19, 20, 21, 22, 24, 25, 26, 27, 28, 29, 30, 33, 34, 35, 36

No information scored as 1, Some limited information scored as 2 and Detailed information scored as 3 for each item. Higher overall scale score indicates more information received during antenatal preparation.

If more than one set of antenatal preparation has been attended, participants should be asked to report on each set and their highest score of information received within any of the programmes utilised. Thus, a composite scale should be created for anyone attending two or more classes, with highest scores for each item of both normality-and broader-focused information used to provide individual sub totals for the two dimensions and then an overall total in analysis.

**Obstetric Complications and Procedures Scale (OCPS)**

| **During the birth of your baby did you experience any of the following complications or procedures?**  **Please tick all that apply** | |
| --- | --- |
| Breaking waters, rupturing membranes artificially |  |
| Waters breaking (naturally or artificially) a prolonged period before going into labour or having contractions |  |
| Starting labour/ Induction of labour by membrane sweep(s) |  |
| Starting labour/ Induction of labour by use of vaginal gel or pessary |  |
| Starting labour/ Induction of labour by use of oxytocin drip |  |
| Speeding up labour/ Augmentation |  |
| Birth assisted by forceps |  |
| Birth assisted by ventouse (method of assisting delivery of your baby using a [vacuum](about:blank) device) |  |
| Episiotomy (surgical incision of the [perineum](about:blank) to enlarge the opening for the baby) |  |
| First- or second-degree perineal tear (tears of the skin and other soft tissue around the vagina) |  |
| Third- or fourth-degree perineal tear (deeper tears of the skin around the vagina that extend to the muscle that controls the anus (the anal sphincter)). |  |
| Baby presenting by breech (bottom first) |  |
| Nuchal cord (cord round baby’s’ neck) |  |
| Baby distressed during labour |  |
| Electronic monitoring of baby throughout labour |  |
| Emergency Caesarean section (When due to an unexpected medical problem, your baby is born through a cut in your lower abdomen to your womb) |  |
| Active management to speed up the delivery of the placenta using an injection |  |
| Retained placenta (The placenta having not been pushed out within an hour of birth, believed to be ‘retained’ and help is required to remove it) |  |
| The need for special care baby unit |  |
| Excessive blood loss after birth |  |
| The need for an extended stay in hospital longer than 3 days following labour for you |  |

| **Scoring Instructions.** **Obstetric Complications and Procedures Scale (OCPS)**  For overall score, add up each item ticked using the individual item weighting. Higher score reflects more Severe and Sudden Obstetric Complications and Procedures during birth. Example as below; Breaking waters, rupturing membranes artificially (2) + Third- or fourth-degree perineal tear (5) + The need for special care baby unit (4) = 11. | | |
| --- | --- | --- |
| Breaking waters, rupturing membranes artificially | (2) | **/** |
| Waters breaking (naturally or artificially) a prolonged period before going into labour or having contractions | (2) |  |
| Starting labour/ Induction of labour by membrane sweep(s) | (2) |  |
| Starting labour/ Induction of labour by use of vaginal gel or pessary | (2) |  |
| Starting labour/ Induction of labour by use of oxytocin drip | (3) |  |
| Speeding up labour/ Augmentation | (3) |  |
| Birth assisted by forceps | (4) |  |
| Birth assisted by ventouse (method of assisting delivery of your baby using a [vacuum](about:blank) device) | (4) |  |
| Episiotomy (surgical incision of the [perineum](about:blank) to enlarge the opening for the baby) | (4) |  |
| First- or second-degree perineal tear (tears of the skin and other soft tissue around the vagina) | (3) |  |
| Third- or fourth-degree perineal tear (deeper tears of the skin around the vagina that extend to the muscle that controls the anus (the anal sphincter)). | (5) | **/** |
| Baby presenting by breech (bottom first) | (3) |  |
| Nuchal cord (cord round baby’s’ neck) | (2) |  |
| Baby distressed during labour | (4) |  |
| Electronic monitoring of baby throughout labour | (2) |  |
| Emergency Caesarean section (When due to an unexpected medical problem, your baby is born through a cut in your lower abdomen to your womb) | (4) |  |
| Active management to speed up the delivery of the placenta using an injection | (2) |  |
| Retained placenta (The placenta having not been pushed out within an hour of birth, believed to be ‘retained’ and help is required to remove it) | (4) |  |
| The need for special care baby unit | (4) | **/** |
| Excessive blood loss after birth | (5) |  |
| The need for an extended stay in hospital longer than 3 days following labour for you | (3) |  |
| **Total Score** | | **11** |

A 2-way random-effects model was chosen to allow reliability results of midwife ratings to be generalised to the midwifery population. In terms of process, it was important for raters to provide scores that were similar in absolute value and so absolute agreement was selected. ICC estimates and their 95% confidence intervals were calculated using SPSS v.25.

| **Supplementary Table 1.** Midwife ratings to create the weighted continuous obstetric procedures and complications scale | | | | | | | | | | | |
| --- | --- | --- | --- | --- | --- | --- | --- | --- | --- | --- | --- |
| **Items** | **Raters** | | | | | | | | | **Mean score** | **Item weighting** |
|  | 1 | 2 | 3 | 4 | 5 | 6 | 7 | 8 | 9 |  |  |
| Rupturing membranes artificially | 1 | 1 | 3 | 4 | 1 | 3 | 2 | 2 | 3 | 2.22 | 2 |
| Waters breaking prolonged period before going labour or contractions | 1 | 1 | 2 | 2 | 1 | 3 | 2 | 5 | 4 | 2.33 | 2 |
| Membrane sweep(s) | 1 | 1 | 3 | 2 | 2 | 3 | 2 | 2 | 1 | 1.89 | 2 |
| Use of vaginal gel or pessary | 1 | 1 | 3 | 3 | 2 | 3 | 3 | 1 | 3 | 2.22 | 2 |
| Oxytocin drip | 1 | 1 | 4 | 4 | 2 | 4 | 3 | 3 | 5 | 3.00 | 3 |
| Augmentation | 1 | 1 | 4 | 5 | 2 | 4 | 3 | 2 | 5 | 3.00 | 3 |
| Forceps | 2 | 3 | 5 | 5 | 5 | 5 | 4 | 5 | 5 | 4.33 | 4 |
| Ventouse | 2 | 3 | 5 | 5 | 4 | 5 | 4 | 5 | 5 | 4.22 | 4 |
| Episiotomy | 2 | 4 | 5 | 5 | 5 | 5 | 4 | 5 | 5 | 4.44 | 4 |
| 1^st^ / 2^nd^ degree perineal tear | 2 | 2 | 4 | 3 | 4 | 5 | 3 | 3 | 4 | 3.33 | 3 |
| 3^rd^ / 4^th^ degree perineal tear | 4 | 4 | 5 | 5 | 5 | 5 | 4 | 5 | 5 | 4.67 | 5 |
| Breech | 2 | 4 | 4 | 1 | 2 | 5 | 3 | 4 | 4 | 3.22 | 3 |
| Nuchal cord | 1 | 1 | 2 | 4 | 3 | 3 | 2 | 3 | 3 | 2.44 | 2 |
| Baby distressed during labour | 3 | 3 | 5 | 5 | 4 | 5 | 4 | 3 | 5 | 4.11 | 4 |
| Electronic monitoring of baby throughout labour | 1 | 1 | 3 | 4 | 1 | 3 | 1 | 2 | 4 | 2.22 | 2 |
| Emergency Caesarean section | 3 | 3 | 5 | 5 | 5 | 5 | 4 | 5 | 5 | 4.44 | 4 |
| Active management of the third stage of labour | 1 | 1 | 3 | 3 | 1 | 2 | 1 | 2 | 4 | 2.00 | 2 |
| Retained placenta | 3 | 3 | 4 | 5 | 2 | 4 | 4 | 3 | 4 | 3.56 | 4 |
| The need for special care baby unit | 4 | 3 | 5 | 5 | 4 | 5 | 4 | 4 | 5 | 4.33 | 4 |
| Excessive blood loss after birth | 3 | 4 | 5 | 5 | 5 | 5 | 4 | 5 | 5 | 4.56 | 5 |
| Extended stay in hospital 3+ days mother | 2 | 2 | 4 | 5 | 1 | 4 | 4 | 3 | 4 | 3.22 | 3 |

**Overall measure of Total Preparation**

| **Supplementary Table 2.** Summary of the birth experience **/** total preparation hierarchical regression model | | | |  |
| --- | --- | --- | --- | --- |
| **Variable** | **B** | **SE B** | **β** | |
| Step 1 |  |  |  | |
| Constant | 13.35 | 0.32 |  | |
| Complications /Procedures | -0.11 | 0.02 | -.39*** | |
| Step 2 |  |  |  | |
| Constant | 11.38 | 0.70 |  | |
| Complications /Procedures | -0.11 | 0.02 | -.38*** | |
| Total Preparation | 0.03 | 0.01 | .18** | |
| Step 3 |  |  |  | |
| Constant | 9.82 | 1.29 |  | |
| Complications /Procedures | -0.01 | 0.07 | -.04 | |
| Total Preparation | 0.04 | 0.02 | .33** | |
| Complications /Procedures * Total Preparation Interaction | -0.00 | 0.00 | -.37 | |
| *R²* = .15 for step 1 (*p*< .001), ∆*R*² = .03 for step 2, ∆*R*² = .01 for step 3 | | | | |
| *Note.* *** p < .001; ** p < .01; * p < .05, Change in *R*²denoted as ∆*R*² | | | |  |

| **Supplementary Table 3.** Summary of the overall emotional experience **/** total preparation model of hierarchical regression | | | |
| --- | --- | --- | --- |
| **Variable** | **B** | **SE B** | **β** |
| Step 1 |  |  |  |
| Constant | 28.13 | 0.90 |  |
| Complications /Procedures | -0.33 | 0.05 | -.39*** |
| Step 2 |  |  |  |
| Constant | 21.95 | 1.98 |  |
| Complications /Procedures | -0.32 | 0.05 | -.38*** |
| Total Preparation | 0.08 | 0.02 | .20** |
| Step 3 |  |  |  |
| Constant | 24.03 | 3.66 |  |
| Complications /Procedures | -0.44 | 0.20 | -.54* |
| Total Preparation | 0.05 | 0.05 | .13 |
| Complications /Procedures * Total Preparation Interaction | 0.00 | 0.00 | .17 |
| *R*² = .16 for step 1 (*p*< .001), ∆*R*² = .03 for step 2, ∆*R*² = .01 for step 3 | | | |
| *Note.* *** p < .001; ** p < .01; * p < .05, Change in *R*² denoted as ∆*R*² | | | |

| **Supplementary Table 4**. Comparison of findings for traumatic birth vs. non-traumatic birth | | | | |
| --- | --- | --- | --- | --- |
|  | No Trauma experienced | | Trauma experienced | |
|  | *Mean* | *SD* | *Mean* | *SD* |
| Normality preparation | 39.67 | 8.72 | 37.57 | 7.72 |
| Broader preparation | 40.22 | 11.10 | 36.89 | 9.44 |
| Total preparation | 79.89 | 18.85 | 74.45 | 16.17 |
| OC&P | 14.15 | 7.88 | 21.09 | 7.75 |
| Total emotions | 25.27 | 6.12 | 18.48 | 6.31 |
| CEQ | 12.49 | 2.01 | 9.83 | 2.22 |
| Complications and procedures | 14.18 | 7.86 | 21.09 | 7.74 |
| PHQ-9 | 5.31 | 4.89 | 9.32 | 6.06 |
| GAD-7 | 4.97 | 4.78 | 9.00 | 5.88 |
| Perceived Social Support | 64.50 | 7.41 | 63.18 | 7.86 |
| Unassisted vaginal delivery (%) | 62 | | 24 | |

| **Supplementary Table 5.** Summary of the trauma experienced / total preparation logistic regression model | | | | | | |
| --- | --- | --- | --- | --- | --- | --- |
|  | |  | | **95% CI for Odds Ratio** | | |
| **Variable** | **B (SE)** | | | **Lower** | Odds ratio | **Upper** |
| Step 1 |  | | |  |  |  |
| Constant | -2.38 (.36) | | |  |  |  |
| OCPS | 0.11*** (.02) | | | 1.08 | 1.12 | 1.16 |
| Step 2 |  | | |  |  |  |
| Constant | -0.99 (.70) | | |  |  |  |
| OCPS | 0.11*** (.02) | | | 1.08 | 1.12 | 1.16 |
| Total Prep | -0.02 * (.01) | | | 0.97 | 0.98 | 1.00 |
| Step 3 |  | | |  |  |  |
| Constant | -3.05 (1.64) | | |  |  |  |
| OCPS | | | 0.23** (.09) | 1.06 | 1.26 | 1.50 |
| Total Prep | | | .01 (.02) | .97 | 1.01 | 1.05 |
| OCPS * Total prep Interaction | | | -.00 (.00) | 1.00 | 1.00 | 1.00 |
| *R²* = .03 (Hosmer & Lemeshow), .15 (Cox & Snell), .21 (Nagelkerke). Model ᵡ^2^(1) = 8.77  *R²* = .01 (Hosmer & Lemeshow), .17 (Cox & Snell), .23 (Nagelkerke). Model ᵡ^2^(1) = 3.28, for step 2,  *R*² = .02 (Hosmer & Lemeshow), .18 (Cox & Snell), .24 (Nagelkerke). Model ᵡ^2^(1) = 5.77, for step 3  *** *p* < .001; ** *p* < .01; * *p* < .05 | | | | | | |

**Impact of the COVID-19 pandemic**

The summary of the themes reported by women on the impact of the pandemic on birth experience are presented in Table 6. In total, 226 participants (90%) reported the pandemic had impacted the antenatal preparation they were able to access. The most prominent theme was the cancellation of classes or classes not running, reported by 107 (%) participants.

For the first question, 251 women responded, with 226 (90%) stating the pandemic had impacted the antenatal preparation they were able to access. The most prominent theme was the cancellation of classes or classes not running, reported by 107 participants. For the second question, 249 responded, with 115 of them (46.2%) stating they were not able to have all the people they had planned to be with them in labour and birth. The most prominent theme was only being able to have one birth partner present, reported by 62 participants.

There were 249 women who responded to question three, with 150 (60.2%) stating they were not able to have someone with them for all the time they had planned during birth. The most prominent theme reported by 54 participants was their partner only being allowed to be present during active labour. Question four was answered by 249 women, of which 170 (68.3%) stated their birth experience was affected in other ways by the COVID pandemic. The most prominent theme was not being able to have visitors or visitors being heavily restricted, reported by 82 participants.

| **Supplementary Table 6.** Summary of the themes reported by women on the impact of the pandemic on birth experience | |
| --- | --- |
| Question | Themes observed (N) |
| 1. Did this impact on what antenatal preparation you were able to access? | Classes cancelled / not running (107) - *8 of whom said they had to pay for private classes instead as their local NHS classes were not running, 3 stating Classes were not available/ not offered.* |
|  | Changes to antenatal appointments (55) *- with 33 reporting attending antenatal appointments alone meant their partner missing out on the experience, and not being able to access the same information. 19 reported antenatal appointments were limited or reduced, meaning women reported less access to information during their appointments, limited time to ask questions, in some cases felt rushed. 4 reported antenatal appointments were over the phone not face to face.* |
|  | Online classes only (47) – *with* *9 stating this limited or gave no opportunity to socialise with other expectant mothers, and 6 stating the online classes provided limited information.* |
|  | Changes to classes (23) –*13 reported Reduced classes, time and number of classes reduced, limited information provided and limited opportunity to ask questions. 10 referenced changing from face to face to online classes.* |
|  | Not able to go on a tour of the maternity unit as originally planned (7) |
| 2. Were you able to have ALL the people you had planned to be with you in labour and birth? | Only one birth partner allowed (62) |
|  | Unable to have mother present (50) |
|  | Unable to have sister present (9) |
|  | Unable to have other (4) - *Doula (2), Mother-in-law (1), No Birth partners (1)* |
|  | Unable to have partner present (3) |
| 3.If someone was with you for the birth were they able to be there for all the time you had planned? | Partner only allowed to be present for Active labour only (54) - *established labour with reports of some birth partners only being allowed into the hospital when birth was imminent.* |
|  | Partner had to leave after birth (51) - *varied from straight away, 10 mins, 2 hours to 4 hours, this was often reported because birth partner were not permitted on the postnatal wards due to COVID-19* *restrictions.* |
|  | Induced alone (29) *- due to visiting being heavily restricted.* |
|  | Partner only allowed to be present once a certain threshold of cervical dilation was met (21) - *this varied in number between 4cm/ 5cm/ 6cm/ 7cm.* |
|  | Partner waiting in the car park/outside (5) - *due to visiting being heavily restricted.* |
| 4.Was your birth experience affected in any other way by the COVID pandemic? | No visitors/ heavily restricted visitors (82) - *this included no other visitors allowed and birth partner visit often limited to active stage of labour and having leave a short time after the birth. - With many women noting their partners or they were unable to leave hospital during labour for food/ drink/ fresh air.* |
|  | Feeling unsupported (30) and alone (20) -*7 of which overlapped with women reporting themes of feeling unsupported by staff and feeling alone.* |
|  | Extra use of PPE/ covid measures (26) - *Including those suspected to be covid positive, having to take covid tests, staff wearing PPE, partner wearing PPE and in some cases mothers wearing masks in the early stages or immediately after giving birth.* |
|  | Partners not able to attend antenatal appointments (20) *- including scans etc, having to go alone – including no or limited No antenatal preparation.* |
|  | Feeling mental health and wellbeing had been impacted (6) - *with reports of low mood (3), worry (1), or anxiety (1) due to impacted experiences.* |
|  | Birth choices limited (6) *– including unable to have a home birth or unable to have a water birth.* |
| *Note.* Multiple themes may have noted within single responses; therefore, numbers do not add up to sample size | |


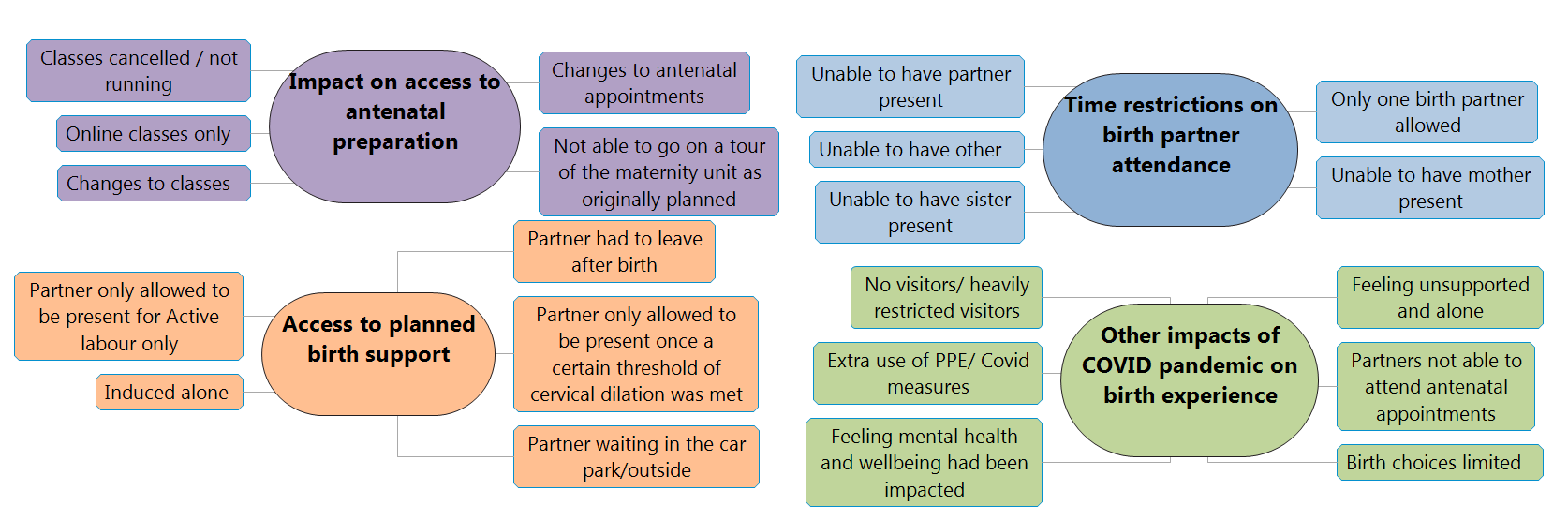


**Supplementary Figure 1.** A visual summary of the four main themes reported by women outlining the impact of the pandemic on birth experience
